# Historical redlining and cardiovascular health: The Multi-Ethnic Study of Atherosclerosis

**DOI:** 10.1073/pnas.2110986118

**Published:** 2021-12-13

**Authors:** Mahasin S. Mujahid, Xing Gao, Loni P. Tabb, Colleen Morris, Tené T. Lewis

**Affiliations:** ^a^Division of Epidemiology, School of Public Health, University of California, Berkeley, CA 94720;; ^b^Department of Epidemiology and Biostatistics, Dornsife School of Public Health, Drexel University, Philadelphia, PA 19104;; ^c^Department of Epidemiology, Rollins School of Public Health, Emory University, Atlanta, GA 30322

**Keywords:** structural racism, redlining, neighborhood, cardiovascular health, MESA

## Abstract

We investigated historical redlining, a government-sanctioned discriminatory policy, in relation to cardiovascular health (CVH) and whether associations were modified by present-day neighborhood physical and social environments. Data included 4,779 participants (mean age 62 y; SD = 10) from the baseline sample of the Multi-Ethnic Study of Atherosclerosis (MESA; 2000 to 2002). Ideal CVH was a summary measure of ideal levels of seven CVH risk factors based on established criteria (blood pressure, fasting glucose, cholesterol, body mass index, diet, physical activity, and smoking). We assigned MESA participants’ neighborhoods to one of four grades (A: best, B: still desirable, C: declining, and D: hazardous) using the 1930s federal Home Owners’ Loan Corporation (HOLC) maps, which guided decisions regarding mortgage financing. Two-level hierarchical linear and logistic models, with a random intercept to account for participants nested within neighborhoods (i.e., census tracts) were used to assess associations within racial/ethnic subgroups (non-Hispanic White, non-Hispanic Black, Hispanic, and non-Hispanic Chinese). We found that Black adults who lived in historically redlined areas had a 0.82 (95% CI −1.54, −0.10) lower CVH score compared to those residing in grade A (best) neighborhoods, in a given neighborhood and adjusting for confounders. We also found that as the current neighborhood social environment improved the association between HOLC score and ideal CVH weakened (*P* < 0.10). There were no associations between HOLC grade and CVH measures or effect modification by current neighborhood conditions for any other racial/ethnic group. Results suggest that historical redlining has an enduring impact on cardiovascular risk among Black adults in the United States.

## Significance

How structural racism contributes to the disproportionate burden of cardiovascular disease risk on minoritized groups in the United States is understudied. This study examined the impact of historical redlining, a government-sanctioned and racially discriminatory policy, and present-day cardiovascular health (CVH). Results suggested that living in historically redlined neighborhoods was associated with CVH only among Black participants and that within this group, living in a neighborhood with better social environment quality weakened but did not fully attenuate this association. These findings suggest that, similar to the institution of slavery, redlining is one manifestation of structural racism that drives health outcomes today. This work underscores the necessity to investigate structural racism as a root cause of racial/ethnic health inequities.

Cardiovascular disease (CVD) remains the leading cause of death and a significant source of racial/ethnic health disparities in the United States (1–3). As a result, the American Heart Association developed its 2020 Strategic Impact Goals to reduce CVD mortality by 20% and improve cardiovascular health (CVH) by 20% for all Americans (4–6). Currently, less than 5% of US adolescents and adults have ideal CVH (1), which underscores the importance of targeted efforts to improve primordial prevention of CVD. Improving neighborhood environments may be essential to these efforts since a strong body of literature has shown that neighborhood built, social, and socioeconomic environments (e.g., healthy food and physical activity resources and safety) are associated with CVD risk factors and mortality (7, 8). Focusing on modern-day neighborhood environments without acknowledging the historical context that shapes these environments ignores the reality of structural racism and its consequences for the CVH of racially and ethnically minoritized populations. Structural racism, which refers to “the totality of ways in which societies foster racial discrimination, through mutually reinforcing inequitable systems (e.g. housing, education, employment, health care, criminal justice), that in turn reinforce discriminatory beliefs, values, and distribution of resources,” (9) is a major driver of racial/ethnic inequities. However, examinations of the role that structural racism plays in shaping the distribution of resources and opportunities across neighborhoods—which has been demonstrated to significantly impact health—are largely missing from the current literature.

One way to capture structural racism is to examine historical discriminatory policies that negatively impacted Black neighborhoods, given the robust evidence that neighborhood context matters for health. Redlining, a practice that became institutionalized through the federal Home Owners’ Loan Corporation (HOLC) security maps created in the 1930s, is one significant policy that influenced current neighborhood conditions (10, 11). The name “redlining” refers to the process of color-coding areas red if they included high concentrations of Black, immigrant, and working-class residents, deeming these areas hazardous and excessively risky for investment (12). HOLC grades prevented residents in these “undesirable” neighborhoods, especially Black residents, from accessing mortgage financing and home ownership. This led to the systematic disinvestment in redlined neighborhoods for decades, contributing to stark inequities in the quality of the physical and social environments in which historically marginalized populations reside in greater proportions (12). Along with other discriminatory housing policies such as deed restrictions and racial covenants, redlining made predominantly Black neighborhoods more susceptible to the negative impacts of subsequent policies and programs, including urban renewal, planned shrinkage, deindustrialization, and White flight (13). Thus, previously redlined areas have been cumulatively affected by a low prevalence of home ownership, uneven economic development, displacement of residents, community disintegration (13), and lack of access to education and economic opportunities (12).

The potential links between historical redlining and cardiovascular outcomes are supported by strong theoretical frameworks and empirical evidence. Ecosocial theory, which posits that social and material contexts affect health through pathways of embodiment, provides a framework for understanding how redlining may impact CVH (14, 15). These pathways become biologically embedded through physiological disruption that may alter multiple systems including metabolic and cardiovascular systems. Moreover, studies have shown that HOLC risk grades are associated with present-day patterns of racial residential segregation, persistent poverty, and income inequality, all of which have been linked to cardiovascular risk factors and outcomes (10, 16–18). Studies have also begun to link historical redlining to select physical health outcomes (preterm birth, asthma hospitalizations, and self-rated health) (19–22); however, no study to date has examined redlining in relation to cardiovascular risk.

Thus, using data from a multiethnic sample of middle-aged adults, we examined associations between historical redlining and ideal CVH. We examined these associations within racial/ethnic subsamples, given that redlining was a racist discriminatory policy that unfairly targeted and disproportionately impacted racial/ethnic minoritized neighborhoods and individuals, especially Black Americans. Due to the historical and upstream nature of our exposure, we also assessed whether associations were modified by present-day indicators of neighborhood physical and social environment, which are features that can be intervened upon to improve CVH. We hypothesized that racially/ethnically minoritized participants residing in historically redlined areas would have lower ideal CVH scores independent of confounders, and that associations would be most pronounced in neighborhoods with poorer physical and social environments.

## Methods

### Study Population.

The Multi-Ethnic Study of Atherosclerosis (MESA) is a prospective study of 6,814 individuals aged 45 to 84 y and free from CVD at baseline (January 2000 to July 2002). Participants of diverse racial and ethnic backgrounds (self-identified as non-Hispanic White, non-Hispanic Black, Hispanic, and non-Hispanic Chinese) were recruited from six sites across the United States (Baltimore City and County, MD; Chicago, IL; Forsyth County, NC; Los Angeles County, CA; New York City, NY; and St. Paul, MN). Additional details on study recruitment and procedures are described elsewhere (23).

This study is restricted to participants who participated in the MESA Neighborhood ancillary study (*n* = 6,191). We further excluded 1,237 participants who resided outside of the HOLC map coverage areas (described below) and 175 participants who were missing information on study covariates, for a final analytic sample of *n* = 4,779 (77% of the full sample). Compared to the full sample of 6,191, those in our analytic sample were more likely to be Chinese and Hispanic and have a lower income (*SI Appendix*, Table S1). The study was approved by the Institutional Review Board at each of the six study site field centers: Wake Forest University School of Medicine, University of Minnesota, Northwestern University, Columbia University, Johns Hopkins University, and University of California, Los Angeles. Written informed consent was obtained from participants upon their arrival at the study clinic (24).

### Study Outcomes.

Our primary outcomes include three ideal CVH summary scores: ideal CVH, ideal CV health behaviors, and ideal CV health factors. We examined ideal CVH using seven indicators (health factors: cholesterol, fasting blood glucose, blood pressure (BP); health behaviors: smoking, body mass index [BMI], physical activity, and diet) and each indicator was categorized as poor, intermediate, or ideal based on established guidelines and criteria (see *SI Appendix*, Table S2) (4). Total cholesterol and blood glucose were ascertained from 75-mL fasting blood samples collected during a clinical examination. BP was measured as the average of the second and third seated BP readings and BMI was assessed using measured height and weight (BMI = height in meters/weight in square kilograms). Smoking was based on self-reported responses to questions on smoking during the past 30 d and lifetime number of cigarettes smoked. Data on physical activity was obtained from the Cross-Cultural Activity Participation Study to estimate the minutes of vigorous and moderate exercise from conditioning, walking, and leisure-time activity during a typical week (25, 26). Diet was measured using a 120-item food frequency questionnaire that assessed the consumption frequency of healthy foods (fruits and vegetables, fish, and whole grains) and unhealthy foods (sugar-sweetened beverages and sodium) in the past year (27, 28). The CVH summary scores (overall, health factors, and health behaviors) were created as the sum of all seven metrics (ideal CVH; range 0 to 14), three health factors (ideal CV health factors; range 0 to 6), and four health behaviors (ideal CV health behaviors; range 0 to 8) (poor = 0, intermediate = 1, and ideal = 2 for each indicator). As secondary outcomes, we examined the six individual indicators of CVH each as binary outcomes (ideal vs. intermediate/poor). We excluded diet from these analyses given the low prevalence of ideal CV diet in this population (*SI Appendix*, Table S2). We also examined continuous versions of BMI, systolic BP (SBP), and diastolic BP (DBP) (*SI Appendix*, Table S3). We added 10 mmHg and 5 mmHg, respectively, for SBP and DBP for those on antihypertensive medications using established methods to reduce bias (29).

### Study Exposure.

Redlining was assessed using digitized HOLC maps from the University of Richmond’s Mapping Inequality Project. HOLC maps of seven cities corresponding to MESA sites (Los Angeles, CA; New York City, NY; Chicago, IL; Saint Paul, MN; Minneapolis, MN; Winston-Salem, NC; and Baltimore, MD) were linked with participant address information. HOLC scores include four levels: A: best, B: still desirable, C: declining, and D: hazardous (30). We geospatially overlaid HOLC grades with 2000 census tracts using ArcGIS. Out of the 1,174 census tracts in the MESA study population, 949 census tracts were contained within or overlapped with the HOLC map coverage areas. We spatially calculated each census tract’s total land area that was contained within or overlapped with the HOLC map coverage areas. Based on the census tract’s percentage of land in each type of HOLC grade, we weighted the grade by the percentage of land mass. This continuous score was then rounded to four categories that corresponded to historical HOLC grades (i.e., HOLC grade A = 1, B = 2, C = 3, and D = 4). For example, if 30% of a census tract’s land is within a B HOLC area, and 50% is within a C HOLC area, then that tract would receive a score of 2.62, which would be rounded to 3, or a C HOLC score:

$$\mathrm{HOLC}\;\mathrm{Score}=\frac{\sum_{i=1}^{n}w_{i}X_{i}}{\sum_{i=1}^{n}w_{i}}$$

where *n* = four categories of HOLC grades, *i* = type of HOLC grade (A, B, C, D), *w_i_* = percentage of census tract land mass that overlaps with HOLC grade *I*, and *X=* HOLC Grade (A = 1, B = 2, C = 3, D = 4).

We also conducted sensitivity analyses using two other exposure assessment methods from previous studies (*SI Appendix*, Table S4). The first method, which we refer to as the “centroid method,” assigns census tracts HOLC grades based on where the centroid of the census tract falls on the HOLC map (22). Although this approach is straightforward, it is limited by the arbitrary nature of using the centroid and many tracts may be excluded if the centroid falls outside of the HOLC map coverage area, even if a large proportion of the tract falls within. The second method, which we refer to as the “land area method,” classifies census tracts based on the proportion of the land area that falls into a HOLC grade (21). This method classifies census tracts that were 50 to 100% in a single HOLC area as the corresponding HOLC risk grade, census tracts with at least 50% area that crosses multiple HOLC areas as “mixed,” and the rest of the census tracts as “unclassified.” The advantage of this method is that it offers a more detailed characterization, with six types of HOLC grade. However, the limitation is that power is reduced due to smaller cells. Our primary approach to assigning HOLC grades can be considered a modified version of the second approach, allowing us to address the aforementioned limitations and potential for exposure misclassification.

### Effect Measure Modifiers.

Neighborhood social and physical environment were included as effect measure modifiers. Neighborhood environments were assessed as part of the MESA Neighborhood ancillary study, which included surveys of MESA participants and non-MESA participants residing in the same neighborhoods. The physical environment was characterized by agreement to statements about the quality of the food environment (three items) and walking environment in the 1 mile surrounding the home (seven items). The social environment was assessed by agreement to statements about the aesthetic quality (five items), safety (three items), and social cohesion (four items) of the neighborhood. Both neighborhood summary measures and subcomponents have been used in prior studies and shown to have good psychometric and ecometric properties (31–33).

### Additional Study Covariates.

The following set of confounders were included in analytic models: age (years), sex (female/male), level of education (bachelor or graduate degree, technical school or associate degree, high school diploma, and less than high school), and family income in the past 12 mo.

### Analyses.

We conducted descriptive analyses to characterize the distribution of all study covariates and to examine bivariate relationships between study covariates and study outcomes, effect measure modifiers, and exposures. To examine race/ethnicity-stratified associations between redlining HOLC grades and ideal CVH measures, we used two-level hierarchical linear models, with a random intercept to account for participants nested within neighborhoods (i.e., census tracts) for each racial–ethnic subgroup. This a priori decision was supported by a statistically significant interaction between HOLC grade and race/ethnicity (*P* < 0.001). It also addresses concerns of limited overlap in study covariates across combinations of race/ethnicity and HOLC grade. To examine race/ethnicity-stratified associations between redlining HOLC grades and the individual components of ideal CVH, we used two-level hierarchical logistic models.

To assess effect measure modification by neighborhood social and physical environments, we included the corresponding cross-product terms in analyses. We used a threshold of *P* < 0.10 to assess the significance of the interaction terms, as is commonly done in tests of statistical interaction in epidemiologic research (34). We conducted sensitivity analyses across three different methods of exposure assessment to ensure the robustness of results. All analyses were conducted in R and ArcGIS.

## Results

Of the 4,779 participants residing in 949 neighborhoods (mean = 5.05 participants per neighborhood), the mean age was 61.9 y (SD = 10.3), 53.6% were female, and the distribution of race/ethnicity was 26.7% Black (*n* = 1,277), 23.9% Hispanic (*n* = 1,142), 13.3% Chinese (*n* = 634), and 35.1% White participants (*n* = 1,726). A greater proportion of Black, Hispanic, and Chinese participants compared to White participants had less than a high school diploma (12.0% Black; 44.0% Hispanic; 24.0% Chinese; 4.6% White), a family income of <$25,000 (32.1% Black, 50.6% Hispanic, 49.7% Chinese; 16.6% White), and lived in neighborhoods with worse physical and social environments (Table 1). Ideal CVH (mean = 8.0 Black, 8.1 Hispanic, 9.7 Chinese, 9.0 White participants) and CV health behaviors (mean = 4.1 Black, 4.1 Hispanic, 5.4 Chinese, 4.7 White participants) were highest in Chinese and lowest in Black and Hispanic participants. The ideal CV health factors summary score was highest in White and lowest in Black participants (mean = 4.0 Black, 4.0 Hispanic, 4.2 Chinese, 4.4 White participants) (Table 1).

**Table 1.** Participant characteristics and average CVH Scores with race/ethnic groups, MESA, 2000 to 2001

	Race/ethnicity																
	Overall *n* = 4,779	Black *n* = 1,277				Hispanic *n* = 1,142				Chinese *n* = 634				White *n* = 1,726			
	*N* (%)	%	Mean CVH	Mean behavjior	Mean health factor	%	Mean CVH	Mean behavior	Mean health factor	%	Mean CVH	Mean behavior	Mean health factor	%	Mean CVH	Mean behavior	Mean health factor
Overall mean			8.0	4.1	4.0		8.1	4.1	4.0		9.7	5.4	4.2		9.0	4.7	4.4
CVH score																	
Sex																	
Female	2,561	56.4	7.9	4.0	3.9	52.5	8.0	4.1	4.0	51.3	9.8	5.5	4.3	53.1	9.1	4.8	4.4
	(53.6)																
Male	2,218	43.6	8.2	4.2	4.0	47.5	8.1	4.1	4.1	48.7	9.5	5.3	4.2	46.9	8.9	4.6	4.3
	(46.4)																
Age (continuous)*, y	61.9	62.4	—	—	—	61.2	—	—	—	62.1	—	—	—	62.2	—	—	—
	(10.3)	(10.1)				(10.4)				(10.2)				(10.4)			
45–54	1,431	29.5	8.3	3.9	4.4	32.6	8.5	3.9	4.7	27.8	10.0	5.1	4.9	29.3	9.4	4.5	4.9
	(29.9)																
55–64	1,315	27.2	7.9	4.0	3.9	27.5	7.9	3.9	4.0	28.2	9.6	5.4	4.3	27.5	9.0	4.6	4.3
	(27.5)																
65–74	1,388	31.2	7.8	4.3	3.6	26.4	7.8	4.2	3.5	30.3	9.5	5.6	3.9	28.7	8.8	4.8	4.0
	(29.0)																
75–84	645	12.1	8.2	4.4	3.8	13.5	7.9	4.5	3.5	13.7	9.5	5.8	3.7	14.4	8.9	5.0	4.0
	(13.5)																
Education																	
Bachelor’s/graduate degree	1,723	33.3	8.5	4.4	4.1	10.6	9.0	4.5	4.6	37.9	9.8	5.5	4.3	54.3	9.6	5.0	4.6
	(36.1)																
Technical/associate	1,325	34.8	8.0	4.0	4.0	25.0	8.3	4.1	4.1	21.5	9.8	5.5	4.3	26.6	8.6	4.4	4.3
	(27.7)																
High school	844	19.9	7.6	3.9	3.7	20.5	7.9	3.9	4.1	16.7	9.6	5.3	4.3	14.5	8.2	4.3	3.9
	(17.7)																
Less than high school	887	12.0	7.4	3.8	3.7	44.0	7.8	4.0	3.8	24.0	9.3	5.3	4.0	4.6	7.6	3.8	3.8
	(18.6)																
Family income																	
>$50,000	1,782	34.6	8.4	4.2	4.2	16.9	8.6	4.2	4.4	26.3	9.8	5.4	4.4	56.8	9.4	4.9	4.5
	(37.3)																
$25,000–50,000	1408	33.3	8.0	4.1	3.9	32.5	8.2	4.0	4.2	24.0	9.7	5.2	4.3	26.7	8.8	4.5	4.3
	(29.5)																
<$25,000	1,589	32.1	7.6	3.9	3.7	50.6	7.8	4.0	3.8	49.7	9.5	5.5	4.2	16.6	8.3	4.3	4.0
	(33.2)																
Social environment*	−0.00	−0.32	—	—	—	−0.46	—	—	—	0.37	—	—	—	0.40	—	—	—
	(1.00)	(1.06)				(0.97)				(0.72)				(0.85)			
Physical environment*	0.02	−0.34	—	—	—	−0.17	—	—	—	0.02	—	—	—	0.43	—	—	—
	(0.99)	(0.88)				(0.66)				(0.56)				(1.21)			
HOLC grade																	
A: best	258	2.8	9.0	4.7	4.4	2.8	8.8	4.7	4.1	8.4	9.7	5.5	4.2	8.0	9.5	5.2	4.3
	(5.4)																
B: still desirable	1,505	32.4	7.8	4.1	3.8	28.0	8.3	4.2	4.1	18.3	9.8	5.4	4.4	37.9	9.0	4.7	4.3
	(31.5)																
C: declining	2,113	41.9	8.0	4.0	4.1	45.9	8.0	4.0	4.0	47.3	9.7	5.4	4.3	43.7	9.0	4.6	4.4
	(44.2)																
D: hazardous	903	22.9	8.0	4.2	3.9	23.8	7.8	4.0	3.9	26.0	9.6	5.4	4.1	10.4	8.8	4.5	4.3
	(18.9)																

*Mean (SD).

Health factor = CV health factor summary score combining the following individual component indicators: cholesterol, glucose, and BP. Overall CVH = sum of seven ideal CV health indicators (cholesterol, fasting blood glucose, BP, smoking, BMI, physical activity, and diet). Health behavior = sum of four ideal CV health behaviors (smoking, BMI, physical activity, and diet). Health factor = sum of three ideal CV health factors (cholesterol, fasting blood glucose, and BP).

Table 1 also shows the distribution of historical HOLC grade overall and by race/ethnicity. Overall, 18.9% of participants lived in historically redlined/hazardous areas (HOLC grade D: “hazardous”), 44.2% lived in declining neighborhoods (HOLC grade C) and the remaining 36.9% lived in desirable areas (HOLC grade A “best” and B “still desirable” neighborhoods). A higher proportion of Black (22.9%), Hispanic (23.8%), and Chinese (26.0%) participants lived in historically redlined areas, compared to White participants (10.4%). HOLC grades also varied by neighborhood physical and social environments with the highest mean physical and social environment scores among those residing in areas historically considered “A: best” neighborhoods and the lowest scores among those residing in “D: hazardous” neighborhoods. The lowest social environment scores were among Black (mean = −1.02) and Hispanic (mean = −0.68) participants residing in historically redlined areas and the best social environments were among Chinese (mean = 1.11) and White participants (mean = 1.25) residing in historically “A: best” neighborhoods (*SI Appendix*, Fig. S1). An examination of CVH scores across HOLC grades revealed nonlinear patterns. Among Black, Hispanic, and White participants, mean CVH scores were highest in those residing in neighborhoods historically considered “A: best” (mean CVH in A: Best neighborhoods = 9.0 for Black, 8.8 for Hispanic, 9.5 for White participants). However, CVH scores were not consistently lowest in historically redlined neighborhoods (Table 1).

In multilevel race/ethnicity-stratified models, adjusted for confounders, HOLC grade was negatively associated with ideal CVH, health behaviors, and health factors among Black participants (Table 2). Black adults who lived in historically redlined areas had a 0.82 (95% CI: −1.54, −0.10) lower CVH score compared to Black adults residing in “A: best” neighborhoods, in a given neighborhood and adjusting for age, sex, education, and income. Black participants residing in areas historically graded as “C: declining” (β = −0.83, 95% CI: −1.53, −0.12) and “B: still desirable” (β = −1.09, 95% CI: −1.80, −0.38) also had a lower CVH score compared to those residing in the “A: best” areas. Similar patterns were observed for ideal CV health factors and health behaviors with the exception that some comparisons included the null. For example, comparisons between “D: hazardous” and “A: best” included the null for ideal CV health behaviors (β = −0.38, 95% CI: −0.91, 0.16) and comparisons between “C: declining” and “A: best” included the null for ideal CV health factors (β = −0.28, 95% CI: −0.70, 0.14). There was no association between HOLC grade and any of the ideal CV health metrics for other racial/ethnic groups.

**Table 2.** Adjusted associations between HOLC grade and ideal CVH summary measures, MESA, 2000 to 2001

	Black (*n* = 1,277)			Hispanic (*n* = 1,142)			Chinese (*n* = 634)			White (*n* = 1,726)		
	Overall CVH	Health behavior	Health factors	Overall CVH	Health behavior	Health factor	Overall CVH	Health behavior	Health factor	Overall CVH	Health behavior	Health factor
B	−1.09	−0.56	−0.55	−0.28	−0.17	−0.14	0.01	−0.08	0.12	−0.25	−0.28	0.06
	(−1.80, −0.38)	(−1.09, −0.03)	(−0.97, −0.12)	(−1.07, 0.51)	(−0.74, 0.41)	(−0.61, 0.33)	(−0.55, 0.58)	(−0.51, 0.34)	(−0.26, 0.51)	(−0.66, 0.16)	(−0.58, 0.02)	(−0.14, 0.27)
C	−0.83	−0.56	−0.28	−0.54	−0.30	−0.24	0.01	−0.05	0.05	−0.14	−0.23	0.13
	(−1.53, −0.12)	(−1.08, −0.03)	(−0.70, 0.14)	(−1.33, 0.24)	(−0.87, 0.27)	(−0.71, 0.23)	(−0.51, 0.52)	(−0.43, 0.34)	(−0.30, 0.40)	(−0.55, 0.27)	(−0.53, 0.08)	(−0.07, 0.34)
D	−0.82	−0.38	−0.45	−0.61	−0.27	−0.34	−0.02	−0.07	0.02	−0.33	−0.32	0.01
	(−1.54, −0.10)	(−0.91, 0.16)	(−0.88, −0.01)	(−1.41, 0.20)	(−0.86, 0.31)	(−0.82, 0.14)	(−0.57, 0.52)	(−0.48, 0.34)	(−0.35, 0.40)	(−0.80, 0.15)	(−0.68, 0.03)	(−0.24, 0.26)

HOLC risk grade: A = best (reference); B = still desirable; C = declining; D = hazardous. Overall CVH = sum of seven ideal CV health indicators (cholesterol, fasting blood glucose, BP, smoking, BMI, physical activity, and diet). Health behavior = sum of four ideal CV health behaviors (smoking, BMI, physical activity, and diet). Health factor = sum of three ideal CV health factors (cholesterol, fasting blood glucose, BP). The 95% CI is displayed in parentheses. Models adjust for age, sex, education, and income.

In our secondary analysis examining the individual components of CVH (Table 3), Black adults who lived in historically redlined areas (“D: hazardous” neighborhoods) had lower odds of ideal CV BP (odds ratio [OR] = 0.22, 95% CI: 0.10, 0.50) and ideal CV BMI (OR = 0.40, 95% CI: 0.18, 0.86) compared to Black adults residing in neighborhoods historically considered “A: best” neighborhoods. Black participants residing in areas historically graded as “C: declining” (OR = 0.33; 95% CI: 0.15, 0.72 for BP; OR = 0.40; 95% CI: 0.19, 0.83 for BMI) and “B: still desirable” (OR = 0.26, 95% CI: 0.12, 0.57 for BP; OR = 0.37, 95% CI: 0.17, 0.77 for BMI) also had a lower odds of ideal CV BP and BMI, respectively, compared to those residing in the “A: best” areas. In models examining HOLC score and continuous BMI and BP, we found that Black participants who lived in historically redlined areas had a 2.01 higher BMI (95% CI: 0.01 to 4.01) and 8.33 (95% CI: 1.02 to 16.66) and 4.89 (95% CI: 1.23, 8.56) higher SBP and DBP, respectively, compared to Black adults residing in neighborhoods historically considered “A: best” neighborhoods, independent of confounders (*SI Appendix*, Table S3). There were no associations between HOLC grade and any other individual CV health factors or behaviors. There were also no associations between HOLC grade and any of the seven individual CV health factors, health behaviors, or continuous BMI, SBP, and DBP among Hispanic, Asian, and White participants, with the exception of ideal CV smoking among White participants (Table 3 and *SI Appendix*, Table S3).

**Table 3.** Adjusted associations between HOLC grade and ideal CVH I, MESA, 2000 to 2001

	Race/ethnicity			
	Black *n* = 1,277 PR [95% CI]	Hispanic *n* = 1,142 PR [95% CI]	Chinese *n* = 634 PR [95% CI]	White *n* = 1,726 PR [95% CI]
Ideal CV BP				
HOLC B	0.26 [0.12–0.57]	1.65 [0.60–4.61]	0.88 [0.43–1.81]	1.21 [0.77–1.90]
HOLC C	0.33 [0.15–0.72]	1.72 [0.62–4.75]	1.15 [0.60–2.21]	1.65 [1.05–2.58]
HOLC D	0.22 [0.10–0.50]	1.41 [0.50–3.98]	1.26 [0.63–2.54]	1.38 [0.81–2.33]
Ideal CV glucose				
HOLC B	0.63 [0.28–1.43]	0.69 [0.28–1.74]	1.86 [0.89–3.87]	0.95 [0.56–1.62]
HOLC C	0.74 [0.33–1.68]	0.52 [0.21–1.30]	1.18 [0.63–2.23]	1.11 [0.65–1.91]
HOLC D	0.66 [0.29–1.52]	0.50 [0.20–1.25]	1.10 [0.56–2.16]	0.79 [0.42–1.48]
Ideal CV cholesterol				
HOLC B	1.10 [0.54–2.22]	0.85 [0.39–1.86]	1.19 [0.58–2.44]	0.93 [0.63–1.38]
HOLC C	1.58 [0.78–3.17]	0.72 [0.33–1.56]	0.94 [0.49–1.80]	0.91 [0.62–1.35]
HOLC D	1.18 [0.57–2.42]	0.69 [0.31–1.52]	0.76 [0.38–1.53]	0.96 [0.60–1.54]
Ideal CV BMI				
HOLC B	0.37 [0.17–0.77]	0.52 [0.21–1.30]	0.93 [0.44–1.93]	0.90 [0.57–1.43]
HOLC C	0.40 [0.19–0.83]	0.45 [0.18–1.13]	0.72 [0.37–1.40]	1.16 [0.73–1.85]
HOLC D	0.40 [0.18–0.86]	0.49 [0.19–1.26]	0.73 [0.36–1.48]	1.07 [0.62–1.84]
Ideal CV physical activity				
HOLC B	1.15 [0.55–2.37]	0.71 [0.29–1.73]	1.46 [0.68–3.10]	0.51 [0.31–0.84]
HOLC C	0.85 [0.41–1.73]	0.57 [0.24–1.39]	1.16 [0.58–2.31]	0.50 [0.31–0.83]
HOLC D	1.42 [0.68–2.99]	0.54 [0.22–1.32]	1.12 [0.53–2.36]	0.56 [0.32–1.00]
Ideal CV smoking				
HOLC B	0.72 [0.24–2.15]	1.15 [0.32–4.16]	0.23 [0.03–2.06]	0.58 [0.26–1.28]
HOLC C	0.59 [0.20–1.74]	1.21 [0.34–4.34]	0.23 [0.03–1.91]	0.55 [0.25–1.21]
HOLC D	0.63 [0.21–1.89]	1.13 [0.31–4.18]	0.23 [0.03–2.05]	0.51 [0.21–1.22]

HOLC risk grade: A = best (reference); B = still desirable; C = declining; D = hazardous. Model adjusts for age, sex, education, and income. Ideal CV diet excluded given the low prevalence in the population. PR: prevalence ratio.

**Fig. 1.**
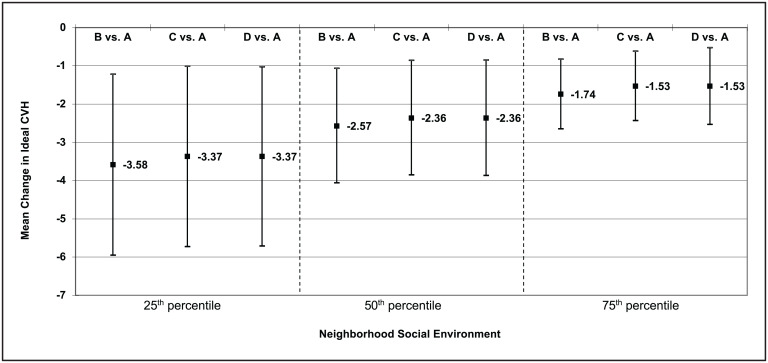
HOLC Risk Grade: A = best; B = still desirable; C = declining; D = hazardous Neighborhood physical environment = summary of 3 neighborhood domains (healthy food, physical activity) Neighborhood social environment = summary of 3 neighborhood domains (aesthetic quality, safety and social cohesion.

We also found that association between HOLC grade and ideal CVH was modified by contemporaneous neighborhood social environment in Black participants (Fig. 1). As the social environment improved, the association between HOLC score and ideal CVH weakened (*P* = 0.085). For example, at the 25th percentile of the distribution (i.e., worse social environment) Black participants who lived in historically redlined areas had a 3.37 (95% CI: −5.71, −1.03) lower CVH score compared to Black participants residing in the “A: best” areas. In contrast, at the 75th percentile of the distribution (i.e., better social environment), Black participants who lived in historically redlined areas had a 1.53 (95% C.I: −2.53, −0.53) lower CVH score compared to Black participants residing in the “A: best” areas. There was no significant interaction between social environment and HOLC grade for the ideal CV health behavior or health factors summary scores and no significant interactions between the physical environment and HOLC grade for all three primary study outcomes among Black participants (all *P* > 0.10). There was also no significant interaction between both neighborhood summary measures and HOLC grade for our three primary study outcomes among other racial/ethnic groups (all *P* > 0.10).

In sensitivity analyses, we found comparable results for both the direction and magnitude of associations. Using the centroid method, we found that associations between HOLC grade and ideal CVH in Black participants was slightly stronger in magnitude, and no associations for other racial/ethnic groups (in Blacks β, 95% CI: −1.64, −2.66, −0.62 for D vs. A; −1.38, −2.39, −0.37 for C vs. A; −1.81, −2.83, −0.80 for B vs. A). Using the land area method, associations were also stronger but included the null for most comparisons among Black participants and there were no associations between HOLC grade and ideal CVH for other racial/ethnic groups (*SI Appendix*, Table S4).

## Discussion

Despite a recent call to action by the American Heart Association acknowledging structural racism as a major driver of racial/ethnic inequities in CVD risk, empirical evidence, especially in relation to historical measures, remains limited (35). In this multiracial/ethnic sample of middle-aged and older adults we examined historical redlining, a discriminatory government-sanctioned policy and an indicator of structural racism, in relation to CVH. In this study, a higher proportion of Black, Hispanic, and Chinese participants lived in historically redlined areas compared to White participants. Moreover, we also found that HOLC grades were associated with all three CVH summary measures: overall CVH, health factors, and health behaviors, as well as with two individual indicators of CVH (BP and BMI), among Black participants. There was no association for Hispanic, Chinese, or White participants. Finally, associations among Black participants were more pronounced among those residing in worse social environments. These results highlight the legacy and lingering impact of historical discriminatory policies on the leading cause of death in the United States.

This study provides empirical support for an association between historical redlining practices and current-day cardiovascular risk. There have been studies that have examined other historical institutions that perpetuated structural racism, such as slavery, in relation to cardiovascular mortality (36, 37). For example, Kramer et al. used national county-level data to document that Black people who lived in counties with the highest concentrations of slaves in 1860 had a 17% slower decline in heart disease mortality compared to the general population from 1968 to 2014 (36). Our study provides empirical evidence that, similar to the institution of slavery, redlining is another manifestation of structural racism whose legacy is driving health disparities today. Research linking these maps with current-day health outcomes is in its infancy, but our results are consistent with a growing body of literature documenting associations between redlining and other health outcomes such as preterm birth and asthma (19, 21, 22).

It is noteworthy that HOLC grade was only associated with CVH among Black participants. Black Americans in the United States have the highest CVD mortality and slowest declines in CVD compared to any other racial/ethnic group (38–40). Although a broad range of social determinants of health have been implicated in this disproportionate burden of CVD, the role of structural racism has been underrepresented in the literature. Historical redlining policies may have a differential impact on Black populations given that at its core redlining was a “racist policy designed to maintain the physical separation of Whites and Black Americans” (12). Moreover, redlining prevented generations of Black families from accumulating wealth from home ownership through the intentional denial of HOLC mortgage loans, leaving predatory loans as the only option. In this study, although a higher proportion of Hispanic and Chinese participants lived in historically redlined areas compared to Black participants, associations between redlining and CVH among these groups were in the hypothesized direction but weaker in magnitude and 95% CIs included the null. Moreover, there was no association among White participants. The null findings in other racial/ethnic groups underscore the importance of measuring and investigating exposures that capture the unique and traumatic nature of the Black experience in the United States (9, 41).

The lack of evidence of a gradient in the association between HOLC grade and cardiovascular outcomes among Black participants contradicted our hypotheses. In fact, the magnitude of association was strongest comparing “B: still desirable” to “A: best” areas for all cardiovascular summary measures. This may be due to higher levels of interpersonal discrimination experienced in more desirable neighborhoods or increases in individual-level psychosocial stressors associated with the cost of upward mobility. For example, Black middle/upper-class Americans may experience more interpersonal discrimination in wealthy, exclusionary neighborhoods, creating stressors that influence CVH. There is extensive evidence linking these psychosocial stressors to a broad range of CVD risk factors and outcomes (42, 43). Alternatively, it could be due to the contemporary neighborhood conditions of previously redlined areas. For example, in our sample, Black participants residing in redlined areas had the worst social environment of any other racial/ethnic and HOLC combination. However, Black participants residing in “still desirable” areas also had worse social environments compared to other racial/ethnic groups residing in “still desirable” areas. Thus, the overall quality of the current neighborhood may be poorer for Black adults, irrespective of a more favorable historical HOLC grade.

In our assessment of the individual CVH component indicators we found associations only among Black participants for BP and BMI such that those residing in historically redlined areas had an 80% lower odds of ideal BP (corresponding to an 8.3 mmHg and 4.9 mmHg higher SBP and DBP when modeled continuously, respectively) and 60% lower odds of ideal BMI (corresponding to a 2.0 higher BMI when modeled continuously) compared to those residing in areas historically graded as “A: best.” BP in particular is a strong predictor of cardiovascular risk, especially among Black Americans (40), and these findings suggest that historical discriminatory policies may have a continued impact on the CVH of Black Americans through elevated BP. The fact that we only found associations with these two cardiovascular risk factors among Black participants is consistent with studies that have found associations between residential segregation and BMI and hypertension among Black Americans (44). Applying a biopsychosocial perspective, living in a previously redlined neighborhood may expose Black residents to more stressors (e.g., lack of access to resources and experiences of discrimination), which can become biologically embodied through mediators such as cortisol, leading to increased BMI (45–48). Similarly, the weathering hypothesis suggests that the cumulative exposure to stress can lead to physiological wear and tear, leading to hypertension (49). We did not find associations between HOLC grade and any of the CVH indicators for other racial/ethnic minoritized groups (i.e., Hispanic and Chinese participants) or for White participants, which further underscores the fact that indicators of structural racism may be particularly salient for Black Americans. Finally, although BMI was assessed as a health behavior based on established criteria to assess ideal CVH, it has multifactorial etiology, and evidence about the relationship between structural racism and BMI can build understanding of how macrolevel factors influence the etiology of CVD risks. Future research is necessary to improve the empirical evidence base of historical indicators of structural racism in relation to other cardiovascular phenotypes.

To further examine the role of current neighborhood conditions, we examined whether associations between HOLC grade and CVH was modified by neighborhood physical and social environment indicators. We found that indeed associations were modified by neighborhood social environment among Black participants such that as the quality of the social environment decreased the magnitude of association between HOLC grade and CVH measures strengthened. However, it is important to note that even among Black participants who resided in areas that currently have a better social environment there were still statistically significant associations between historical HOLC grade and CVH. The lack of modification by physical environment is consistent with the literature documenting that social environment indicators may have a stronger effect on CVH than physical environment indicators for in Black cohorts, including the Jackson Heart Study and the Morehouse‐Emory Center for Health Equity Study (50–52). These findings suggest that a focus on place-based interventions that improve social disorder and neighborhood decay may help lessen but not completely mitigate the impact of structural racism on CVH among Black participants. Moreover, future studies should also examine the potential threat of gentrification, given recent studies documenting that this sociopolitical process may also be more detrimental in Black adults (53).

A few limitations warrant comment. First, we limited our sample to participants who resided in HOLC map coverage areas, which may have introduced selection bias. This, along with the other exclusion criteria, created an analytic sample that had a slightly different sociodemographic profile than the full MESA sample. Second, there is no gold standard for defining HOLC grades using historical city maps that imperfectly aligned with administrative boundaries such as census tracts often used in studies of neighborhood health effects. Although we found results comparable across three leading approaches to classifying HOLC grades, exposure misclassification is still possible. Third, there may be residual confounding given the potential for measurement error of the confounders we adjusted for and an omission of other key confounders, especially those tied to historical correlates of HOLC grades that may also be associated with current-day CVH (e.g., historical neighborhood socioeconomic indicators). Fourth, we may have been underpowered to detect associations between redlining and CVH, especially with individual components of CVH, within all racial and ethnic groups. Only 5% of the sample overall lived in “A: best” areas, representing fewer than 50 Black adults in stratified models. Thus, although we chose to use “A: best” neighborhoods as the reference group to preserve and accurately capture the historical nature of redlining practices and we were able to detect significant associations for Black individuals while achieving good precision around our statistical estimates, our findings should be interpreted with care given that they were based on a less-than-ideal number of participants. Fifth, given that individuals who had clinical CVD were excluded from participation in the study, our findings may be an underestimation of the true association, given that CVD has an earlier onset in many socially marginalized populations who might be most impacted by structural racism. Finally, although the MESA study is a national study, it is limited to six sites and was not designed to be representative of all neighborhoods in those sites, which limits the generalizability of our study findings.

In summary, our findings provide support for the lasting impact of historical discriminatory policies on the current-day high cardiovascular risk among Black adults in the United States. This research underscores the critical importance of empirical investigations of structural racism as root causes of racial/ethnic inequities. Future research should continue to examine discriminatory policies and other indicators of structural racism, including mass incarceration and police violence, on the health of racially minoritized populations.

## Supplementary Material

Supplementary File

## Data Availability

Researchers trained in human subject research may find information on requesting access to the data used in this study at https://mesa-nhlbi.org.
