# Supplementary material for: Historical redlining and cardiovascular health: The Multi-Ethnic Study of Atherosclerosis
Source: Proc Natl Acad Sci U S A. 2021 Dec 13;118(51):e2110986118. doi: 10.1073/pnas.2110986118 (PMC8713797; doi:10.1073/pnas.2110986118)
Supplement: Supplementary File [file pnas.2110986118.sapp.pdf]

Supplemental Table 1. Comparison of Full Sample and Analytic Sample (Multi-Ethnic Study of Atherosclerosis 2000-2001)

	Full Sample (N=6191)	Analytic Sample (N=4779)	p-value
	%	%	
Age (mean, SD)	61.91 (10.1)	61.82 (10.3)	0.646
Sex			
Female	52.4	53.6	0.236
Male	47.6	46.4	
Race			
Black	27.3	26.7	<0.001
Hispanic	21.6	23.9	
Asian	11.8	13.3	
White	39.3	36.1	
Education			
Bachelor's/Grad Degree	36.6	36.1	0.103
Tech school/Associate	28.4	27.7	
High school	18.2	17.7	
Less than High School	16.8	18.6	
Income			
>50K	40.8	37.3	<0.001
25,000-50,000	29.2	29.5	
<25K	30.0	33.2	
Cardiovascular Health, mean summary scores (SD)			
Overall CVH	8.6 (2.1)	8.6 (2.1)	0.288
Health Behaviors	4.1 (1.5)	4.5 (1.5)	0.902
Health Factors	3.7 (1.1)	4.2 (1.3)	0.217

Overall CVH=sum of 7 ideal CV health indicators (cholesterol, fasting blood glucose, blood pressure, smoking, body mass index, physical activity, and diet)
Health Behaviors=sum of 4 ideal CV health behaviors (smoking, body mass index, physical activity, and diet)
Health Factors=sum of 3 ideal CV health factors (cholesterol, fasting blood glucose, blood pressure)

Supplemental Table 2. Indicators of Ideal Cardiovascular Health and Prevalence in Study Population

Component	Score	Definition	% of MESA Participants (N=4779)
**Health Factor**			
**Cholesterol**			
	Poor	≥ 240 mg/dL	440 (9.2)
	Intermediate	200-239 mg/dL or treated to < 200 mg/dL	2068 (43.4)
	Ideal	<200 mg/dL, unmedicated	2259 (47.4)
**Glucose**			
	Poor	≥ 126 mg/dL fasting	380 (8.0)
	Intermediate	100-125 mg/dl fasting unmedicated or treated to <100mg/dL	843 (17.7)
	Ideal	<100mg/dL fasting, unmedicated	3542 (74.3)
**BP**			
	Poor	SBP ≥ 140 mmHg or DBP ≥ 90 mmHg	1159 (24.3)
	Intermediate	SBP 120-139 mmHg or DBP 80-89 mmHg	1937 (40.6)
	Ideal	SBP < 120 mmHg and DBP < 80 mmHg	1680 (35.2)
**Health Behavior**			
**BMI**			
	Poor	≥ 30 kg/m^2	1518 (31.8)
	Intermediate	25 - 29.9 kg/m^2	1844 (38.6)
	Ideal	< 25 kg/m^2	1417 (29.7)
**Physical Activity**			
	Poor	no exercise	1098 (23.0)
	Intermediate	1-149 minutes of moderate or 1-74 minutes of vigorous exercise/week	836 (17.5)
	Ideal	150+ minutes of moderate or 75+ minutes of vigorous exercise/week	2843 (59.5)
**Smoking**			
	Poor	Current Smoker	619 (13.0)
	Intermediate	Former Smoker, quit ≤ 12 months ago	87 (1.8)
	Ideal	Never Smoker or quit > 12 months ago	4073 (85.2)
**Diet**			
	Poor	0-1 components of healthy diet	2700 (58.7)
	Intermediate	2-3 components of healthy diet	1884 (40.9)
	Ideal	4-5 components of healthy diet	17 (0.4)

Supplemental Table 3: Adjusted Associations between HOLC Grade, BMI, and Blood Pressure, Multi-Ethnic Study of Atherosclerosis, 2000–2001

	Black (N=1277)			Hispanic (N=1142)			Chinese (N=634)			White (N=1726)		
	BMI	Systolic BP	Diastolic BP	BMI	Systolic BP	Diastolic BP	BMI	Systolic BP	Diastolic BP	BMI	Systolic BP	Diastolic BP
HOLC B	2.25<br>(0.30, 4.22)	9.18<br>(1.50, 16.89)	4.68<br>(1.10, 8.29)	0.78<br>(-1.19, 2.74)	0.42<br>(-7.36, 8.21)	1.17<br>(-2.62, 4.95)	0.18<br>(-.98, 1.34)	-1.22<br>(-7.93, 5.49)	1.67<br>(-1.71, 5.06)	-0.14<br>(-1.09, 0.80)	-3.85<br>(-7.76, -0.03)	-1.74<br>(-3.60, 0.07)
HOLC C	1.80<br>(-0.14, 3.75)	6.89<br>(-0.71, 14.52)	4.10<br>(0.54, 7.67)	1.10<br>(-0.86, 3.06)	-1.25<br>(-8.99, 6.48)	-0.53<br>(-4.29, 3.23)	0.28<br>(-0.77, 1.34)	-2.16<br>(-8.21, 3.89)	-0.79<br>(-3.84, 2.26)	-0.35<br>(-1.31, 0.60)	-4.64<br>(-8.75, -0.69)	-2.59<br>(-4.51, -0.76)
HOLC D	2.01<br>(0.01, 4.01)	8.83<br>(1.02, 16.66)	4.89<br>(1.23, 8.56)	1.36<br>(-0.63, 3.37)	0.90<br>(-7.03, 8.82)	-0.18<br>(-4.03, 3.67)	0.32<br>(-0.82, 1.46)	-5.06<br>(-11.53, 1.41)	-1.25<br>(-4.51, 2.01)	0.25<br>(-0.93, 1.38)	-3.69<br>(-8.25, 0.82)	-1.64<br>(-3.84, 0.57)

HOLC Risk Grade: A=best (referent); B=still desirable; C=declining; D=hazardous

BMI is continuous BMI; systolic and diastolic blood pressure were adjusted for medication use

95% confidence interval displayed in parenthesis

Models adjust for age, sex, education, and income

Supplemental Table 4: Adjusted Associations between HOLC Grade and Ideal Cardiovascular Health Measures, Multi-Ethnic Study of Atherosclerosis, 2000–2001

	Black (N=1277)			Hispanic (N=1142)			Chinese (N=634)			White (N=1726)		
	Overall CVH	Health Behaviors	Health Factors	Overall CVH	Health Behavior	Health Factor	Overall CVH	Health Behavior	Health Factor	Overall CVH	Health Behavior	Health Factor
**Centroid**												
HOLC B	-1.81 (-2.83, -0.80)	-0.69 (-1.48, 0.10)	-1.14 (-1.75, -0.53)	0.02 (-0.80, 0.84)	0.00 (-0.60, 0.60)	-0.07 (-0.38, 0.53)	-0.07 (-0.86, 0.72)	-0.29 (-0.85, 0.27)	0.22 (-0.30, 0.74)	0.25 (-0.23, 0.74)	0.10 (-0.28, 0.47)	0.09 (-0.10, 0.29)
HOLC C	-1.38 (-2.39, -0.37)	-0.63 (-1.41, 0.15)	-0.74 (-1.35, -0.13)	-0.42 (-1.23, 0.39)	-0.33 (-0.93, 0.26)	-0.03 (-0.47, 0.41)	-0.28 (-0.99, 0.42)	-0.31 (-0.80, 0.18)	0.02 (-0.44, 0.49)	0.11 (-0.37, 0.58)	0.02 (-0.35, 0.39)	0.07 (-0.13, 0.26)
HOLC D	-1.64 (-2.66, -0.62)	-0.60 (-1.39, 0.19)	-1.03 (-1.65, -0.42)	-0.22 (-1.05, 0.62)	-0.11 (-0.71, 0.50)	-0.05 (-0.52, 0.41)	-0.10 (-0.84, 0.64)	-0.17 (-0.70, 0.35)	0.06 (-0.42, 0.55)	-0.01 (-0.54, 0.53)	0.00 (-0.41, 0.41)	-0.07 (-0.31, 0.18)
**Land area**												
HOLC B	-2.03 (-4.03, -0.04)	-0.71 (-2.19, 0.77)	-1.34 (-2.57, -0.12)	-0.59 (-1.61, 0.41)	-0.24 (-0.98, 0.49)	-0.40 (-1.01, 0.20)	-0.04 (-0.86, 0.92)	-0.08 (-0.66, 0.61)	0.07 (-0.52, 0.67)	-0.10 (-0.70, 0.49)	-0.18 (-0.63, 0.26)	0.04 (-0.27, 0.36)
HOLC C	-1.79 (-3.78, 0.20)	-0.76 (-2.23, 0.72)	-1.02 (-2.25, 0.20)	-0.92 (-1.92, 0.07)	-0.45 (-1.17, 0.27)	-0.50 (-1.10, 0.09)	-0.35 (-1.12, 0.42)	-0.05 (-0.71, 0.41)	-0.20 (-0.72, 0.32)	-0.26 (-0.87, 0.32)	-0.25 (-0.70, 0.19)	0.02 (-0.30, 0.33)
HOLC D	-1.89 (-3.88, 0.11)	-0.64 (-2.12, 0.84)	-1.24 (-2.47, -0.01)	-0.86 (-1.88, 0.15)	-0.36 (-1.09, 0.38)	-0.53 (-1.15, 0.07)	-0.27 (-1.07, 0.53)	-0.07 (-0.64, 0.53)	-0.24 (-0.78, 0.31)	-0.32 (-0.97, 0.31)	-0.25 (-0.73, 0.22)	-0.09 (-0.44, 0.26)
Mixed	-1.63 (-3.65, 0.40)	-0.57 (-2.06, 0.94)	-1.06 (-2.30, 0.19)	-1.11 (-2.21, -0.02)	-0.61 (-1.42, 0.19)	-0.51 (-1.13, 0.13)	-0.54 (-1.59, 0.52)	-0.08 (-1.07, 0.46)	-0.21 (-0.93, 0.51)	-0.33 (-0.94, 0.29)	-0.37 (-0.84, 0.09)	0.06 (-0.25, 0.38)
No Grade	-2.03 (-4.02, -0.04)	-0.73 (-2.20, 0.74)	-1.30 (-2.52, -0.07)	-1.03 (-2.04, -0.03)	-0.46 (-1.19, 0.27)	-0.58 (-1.19, 0.02)	-0.17 (-0.93, 0.60)	-0.05 (-0.54, 0.57)	-0.20 (-0.72, 0.32)	-0.64 (-1.20, -0.08)	-0.51 (-0.93, -0.09)	-0.13 (-0.44, 0.18)

Overall CVH=sum of 7 ideal CV health indicators (cholesterol, fasting blood glucose, blood pressure, smoking, body mass index, physical activity, and diet)
HOLC Risk Grade: A=best; B=still desirable; C=declining; D=hazardous
Health Behavior=sum of 4 ideal CV health behaviors (smoking, body mass index, physical activity, and diet)
Health Factor=sum of 3 ideal CV health factors (cholesterol, fasting blood glucose, blood pressure)
95% confidence interval displayed in parenthesis
Models adjust for age, sex, education, income

Supplemental Figure 1: Current Day Mean Neighborhood Physical and Social Environment Scores by Historical HOLC Grade and Race/Ethnicity, The Multi-Ethnic Study of Atherosclerosis 2000-2001

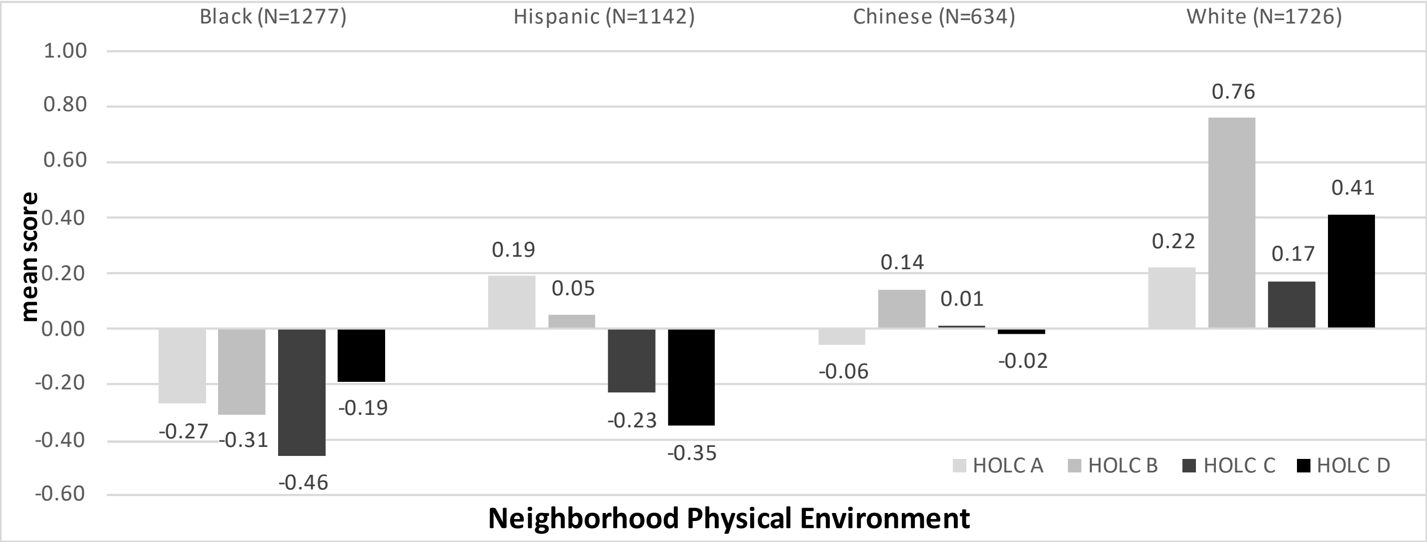

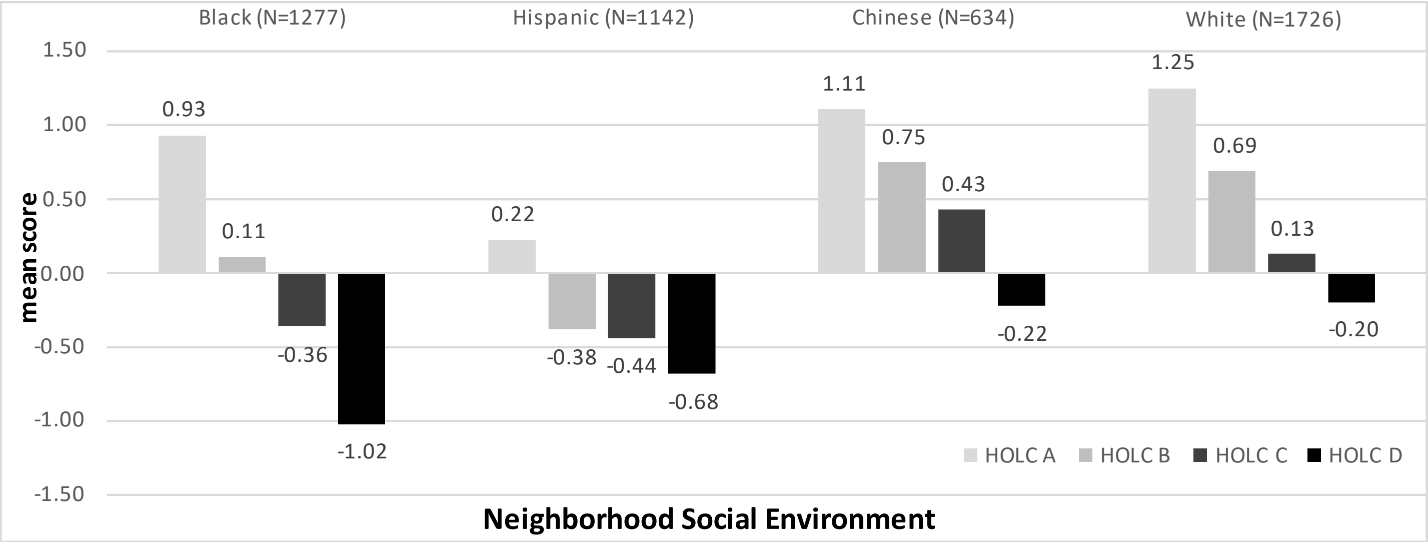

HOLC Risk Grade: A=best; B=still desirable; C=declining; D=hazardous
Neighborhood physical environment=summary of 3 neighborhood domains (healthy food, physical activity)
Neighborhood social environment=summary of 3 neighborhood domains (aesthetic quality, safety and social cohesion
